# Eucaine Lactate

**Published:** 1905-05-27

**Authors:** 


### EUCAINE LACTATE.

There are certain well-recognised drawbacks to the use of cocaine as a local anæsthetic which have rendered it very desirable that some substitute should be found. When β-eucaine hydrochloride was brought out it was at first thought to have so many advantages over cocaine as to quite supplant it in practical work. But it was soon found that β-eucaine was not perfect. Its slight solubility in water, and the marked hyperæmia which followed its local application, were distinct hindrances to its use in a number of cases. Consequently the medical profession had to return once more to an attitude of hopeful expectancy that a really good, local anæsthetic agent would be discovered. It appears that such a substance has now been found. Professor Langgaard, who for some time had been searching for a good substitute for cocaine and β-eucaine hydrochloride, discovered that β-eucaine lactate has certain indisputable advantages over either. Thus, it has not the toxicity of cocaine, it is freely soluble in water (25 per cent. at ordinary temperatures), it does not produce local hyperæmia or ischæmia, and it may be boiled without losing its potency, while its anæsthetic properties are all that can be desired. If it be expedient to produce ischæmia together with anæsthesia, as, for example in certain genito-urinary operations, this can be accomplished readily by the addition of adrenalin to the solution used. It therefore seems probable that β-eucaine lactate will almost entirely take the place hitherto occupied by cocaine and β-eucaine hydrochloride. We have tested samples of the drug obtained from Messrs. A. and M. Zimmerman, and have found it very satisfactory in every way.

